# Synthetic amorphous silica: MAK Value Documentation, addendum – Translation of the German version from 2023

**DOI:** 10.34865/mb763186e10_4ad

**Published:** 2025-12-22

**Authors:** Andrea Hartwig, MAK Commission

**Affiliations:** 1 Institute of Applied Biosciences. Department of Food Chemistry and Toxicology. Karlsruhe Institute of Technology (KIT) Adenauerring 20a, Building 50.41 76131 Karlsruhe Germany; 2 Permanent Senate Commission for the Investigation of Health Hazards of Chemical Compounds in the Work Area. Deutsche Forschungsgemeinschaft, Kennedyallee 40, 53175 Bonn, Germany. Further information: Permanent Senate Commission for the Investigation of Health Hazards of Chemical Compounds in the Work Area | DFG

**Keywords:** synthetic amorphous silica, lung, inflammation, MAK value, maximum workplace concentration, peak limitation, developmental toxicity, genotoxicity, Luft, Luft, Luft, Luft, Luft, Luft, Luft, air, air, air, air, air, air, air

## Abstract

The German Commission for the Investigation of Health Hazards of Chemical Compounds in the Work Area (MAK Commission) has re-evaluated the occupational exposure limit value (maximum concentration at the workplace, MAK value) of synthetic amorphous silica [7631-86-9] considering all toxicological end points. Relevant studies were identified from a literature search and also unpublished study reports were used. The critical effects are the inflammatory effects in the lungs. A NOAEC cannot be established for these effects. In a 90-day study, one form of nanoscale synthetic amorphous silica induced inflammatory effects in the lungs of rats at the lowest concentration tested of 0.5 mg/m^3^ and above. Based on this LOAEC, a maximum concentration at the workplace (MAK value) of 0.02 mg/m^3^ has been derived for the respirable fraction and the substance has been classified in Peak Limitation Category II with an excursion factor of 8. The NOAEL for developmental or perinatal toxicity in rats was 1000 mg/kg body weight and day after gavage; this corresponds to concentrations of 1750 or 2450 mg/m^3^ at the workplace. As the margins between these values and the MAK value are sufficiently large, synthetic amorphous silica has been assigned to Pregnancy Risk Group C. Studies in animals did not show a carcinogenic potential of synthetic amorphous silica, which is relevant for humans. Synthetic colloidal amorphous silica is not mutagenic in vitro or in vivo. The DNA strand breaks and micronuclei that were observed in vitro were not confirmed by the results in vivo. Synthetic colloidal amorphous silica is not absorbed through the skin in toxicologically relevant amounts and there is no evidence that it induces contact sensitization.

**Table:** 

**MAK value (2022)**	**0.02 mg/m^3^ R (respirable fraction)**
**Peak limitation (2021)**	**Category II, excursion factor 8**
	
**Absorption through the skin**	**–**
**Sensitization**	**–**
**Carcinogenicity**	**–**
**Prenatal toxicity (1994)**	**Pregnancy Risk Group C**
**Germ cell mutagenicity**	**–**
	
**BAT value**	**–**
	
CAS number	7631-86-9, 112945-52-5, 112926-00-8, 61790-53-2, 63231-67-4

Documentation for amorphous silica was published in 1989 (Henschler 1991). Cited unpublished toxicological studies from companies have been made available to the Commission.

## General Characteristics

Synthetic amorphous silica (synthetic amorphous silicon dioxide; CAS No. 7631-86-9 for silicon dioxide) includes silicas produced in wet processes such as precipitated silica, colloidal silica or silica gel, as well as pyrogenic silica produced in thermal processes (CAS No. 112945-52-5). This documentation is valid also for uncalcined diatomaceous earth (CAS No. 61790-53-2). Many modified forms exist, some of which have been assigned individual CAS numbers. Synthetic amorphous silica consists of nanosized particles and occurs in both hydrated and non-hydrated forms. These SiO_2_ modifications are X-ray amorphous and are made up of silicon–oxygen tetrahedra. Nanosized amorphous silicas are used as additives in cement, paint, cosmetics or food and as drug delivery devices in the medical sector, among other applications (Maser et al. 2015).

## 1 Toxic Effects and Mode of Action

After exposure of rats by inhalation for 13 weeks, synthetic amorphous silica in concentrations of 0.5 mg/m^3^ and above induced inflammatory changes in the lungs, changes in the bronchoalveolar lavage fluid (BALF) and goblet cell hyperplasia in the nasal cavity.

The substance is not irritating to the eyes, the upper respiratory tract or skin. The substance did not induce sensitizing effects on the skin in an animal study. There are no data for sensitizing effects on the airways.

No toxic effects on development or effects that affected fertility were observed.

The DNA strand breaks and micronuclei that were induced by amorphous silica nanoparticles in vitro were not confirmed by the findings from animal studies.

There is no reliable evidence suggesting that amorphous silica causes carcinogenic effects.

## 2 Mechanism of Action

There are no data for the mechanism underlying the inflammatory effects in the lungs.

## 3 Toxicokinetics and Metabolism

### 3.1 Absorption, distribution and elimination

Compared with crystalline silicon dioxide, amorphous silica is less toxic and is cleared from the lungs more rapidly (IARC 1997). After inhalation exposure to colloidal synthetic amorphous silica (SAS) for 4 weeks in concentrations of 50 and 150 mg/m^3^, half-lives of 40 and 50 days, respectively, were determined in the lungs of rats (Lee and Kelly 1992). Therefore, clearance from the lungs was not yet impaired under these conditions. In comparison, cristobalite (a high-temperature modification of silicon dioxide) was found to have a half-life of more than 125 days in the lungs of rats (Hemenway et al. 1990). After exposure of rats for 5 days to quartz (Min-U-Sil quartz) in a concentration of 50 mg/m^3^, only 20% had been cleared from the lungs after 20 days (Driscoll et al. 1991).

#### Dermal application

As the substance is inorganic, a log K_OW_ has not been determined. The US EPA used models to obtain a log K_OW_ of 0.53 (ECHA 2021). The data for its solubility in water are not consistent. The REACH dossier includes 16 studies that investigated the solubility of different silicon dioxides in water. The highest reported level of solubility was 140 mg/l (ECHA 2021). On the basis of these data and a molar mass of 60.08 g/mol, absorbed amounts of 4 and 0.7 mg, respectively, were calculated using the model of Fiserova-Bergerova et al. (1990) and the IH SkinPerm model of Tibaldi et al. (2014) assuming standard conditions (exposure for 1 hour, surface area of 2000 cm^2^ of skin). In a study in rabbits, the silicon dioxide concentrations in the urine, blood, kidneys, liver and spleen were not increased after dermal exposure to a dose of 10 000 mg/kg body weight (no other details; ECHA 2021).

### 3.2 Metabolism

Amorphous silica probably does not undergo metabolism.

## 4 Effects in Humans

The epidemiological studies included in the 1989 documentation did not find evidence of an association between exposure to amorphous silica and respiratory symptoms (Henschler 1991).

An epidemiological cross-sectional study investigated a possible relationship between exposure to fumed and precipitated synthetic amorphous silica at the workplace and pulmonary effects. The study included 462 male workers from 5 production plants in Germany. At the time of the study, the workers were on average 41 years of age. Of the participants, 76.4% were smokers or former smokers. The average length of exposure was 12.4 years with a maximum duration of 42 years. Exposure levels were determined at different workstations and for different activities. Two different methods were used to determine and evaluate the cumulative inhalation exposure of 484 male workers with an average age of 39.5 (19.5–60.8) years. In the first method, 4 exposure categories were established for each production site. These were defined by in-house experts based on individual work histories: low (< 1 mg/m^3^), moderate (1–4 mg/m^3^), high (4–10 mg/m^3^) and peak (> 10 mg/m^3^). In the second method, job categories were assigned based on individual work histories and similar exposure categories were established for the 5 production sites in order to estimate the level of exposure in each job category retrospectively. Also taken into consideration were the levels of inhalable SAS dust that were recently determined by personal monitoring and detailed information relating to changes that had taken place within the company. A mean cumulative exposure of 56.9 mg/m^3^ × years was estimated using the first method. A geometric mean for exposure of 31.95 mg/m^3^ × years was calculated using the second method. The authors noted that both methods have shortcomings that need to be taken into consideration when evaluating the epidemiological studies (Morfeld et al. 2014). Three exposure categories were established: ≤ 40, > 40 to ≤ 100, > 100 mg/m^3^ × years. None of the participants had pneumoconiosis or fibrosis. The results of a spirometry test were in the normal range for 80% of the workers, while 17% had obstructive findings and 4% restrictive findings. According to the authors, this distribution is about equivalent to that reported by the NHANES III study for white male Americans. The forced vital capacity (FVC) of the test persons revealed a slight, but noticeable, decline by 48 ml at a cumulative exposure level of 80 mg/m^3^ × years (Taeger et al. 2016 a). However, because the method used to collect the exposure data was not precise, the study is not suitable for the derivation of a NOAEC (no observed adverse effect concentration).

## 5 Animal Experiments and in vitro Studies

### 5.1 Acute toxicity

#### 5.1.1 Inhalation

In an inhalation study, groups of 3 male and 3 female Sprague Dawley rats were exposed nose-only for 4 hours to an aerosol of precipitated silica (Zeosil Premium 200 MP; mass median aerodynamic diameter (MMAD) 0.975 µm) in concentrations of 0 or 5.01 mg/m^3^. No signs of acute toxicity were observed within the observation period of 14 days. The study was carried out according to OECD Test Guideline 436 (ECHA 2021).

#### 5.1.2 Oral administration

Groups of 5 male and 5 female Wistar rats were given single gavage doses of fumed silica (Cab-O-Sil) of 0 or 5000 mg/kg body weight. No symptoms were observed in the 14-day observation period. Therefore, the oral LD_50_ is above 5000 mg/kg body weight in rats (ECHA 2021). Other studies found the LD_50_ to be above 1000 mg/kg body weight after the administration of oral doses of silica of different trade names (ECHA 2021).

#### 5.1.3 Dermal application

The dermal LD_50_ for Syloid 244 was above 2000 mg/kg body weight in rabbits. No skin irritation was observed (ECHA 2021). The dermal LD_50_ for silicon dioxide nanoparticles without surface functionalization (Zeo49) was above 5000 mg/kg body weight in rabbits (ECHA 2021).

#### 5.1.4 Intraperitoneal and intravenous injection

ICR mice were given a single intraperitoneal injection of amorphous silica with an average particle size of 12 nm in doses of 0, 50, 100 or 250 mg/kg body weight. The sex of the animals was not specified. The animals were examined 3 days after exposure. The increased activation of peritoneal macrophages and the elevated expression of genes that contribute to the formation of inflammation-promoting cytokines (interleukin (IL)-1, IL-6, TNF-α) or enzymes (iNOS, COX-2) were observed at the lowest dose tested and above. The authors did not provide any further data to characterize the silica that was tested (Park and Park 2009).

Male Wistar rats were given 3 consecutive intravenous injections of amorphous silica in doses of 0, 25 or 50 mg/kg body weight (particle size 15 nm; Levasil^®^ 200/40) or 0, 25, 50 or 125 mg/kg body weight (particle size 55 nm; Levasil^®^ 50/50) 48, 24 and 4 hours before sacrifice. In the group of animals exposed to 15-nm particles of amorphous silica in a dose of 50 mg/kg body weight, slight histopathological changes and a slight increase in the infiltration of neutrophilic leukocytes and mononuclear cells were observed in the liver. Additionally, the proinflammatory cytokines TNF-α and IL-6 were increased in the plasma of the animals that were exposed to the 15-nm particles at a dose of 50 mg/kg body weight and to the 55-nm particles at a dose of 125 mg/kg body weight (Downs et al. 2012).

Mice were injected once intravenously with a suspension of amorphous silica particles of various sizes that were labelled with a fluorescent dye. The injected dose was equivalent to 50 mg/kg body weight. The sex of the animals was not specified. The particles were of medium size with diameters of 50, 100 or 200 nm. The animals were examined after 12, 24, 48 and 72 hours or 7 days. After 12 hours, the histological examination showed that the 100-nm and 200-nm particles transiently increased inflammatory responses in the liver in the form of multifocal inflammatory cell foci. The responses were no longer noticeable after 24 hours. All particles were excreted with the bile and the urine. Particle-loaded macrophages were found in the liver and spleen up to 4 weeks after injection (Cho et al. 2009).

#### 5.1.5 Intratracheal instillation

Male rats (Crl:CD (SD)IGS BR) were exposed to precipitated silica (Zeofree 80) by intratracheal instillation at dose levels of 0, 1 or 5 mg/kg body weight. The particles were 90 to 500 nm in diameter. The BALF was examined after 24 hours, 1 week, and 1 and 3 months. At the examination 24 hours after exposure, the silica had induced a reversible inflammatory response in the lungs that was manifest in the form of an increase in neutrophils in the BALF even at the low dose of 1 mg/kg body weight and above. In addition, an increased level of lactate dehydrogenase (LDH) activity in the BALF at doses of 1 mg/kg body weight and above suggests cell damage in the lungs (Sayes et al. 2007).

In another study, a suspension of ultrafine amorphous silica particles with a primary particle diameter of 14 nm was administered once to male A/J mice (5 per group) in doses of 0, 2, 10 or 50 mg/kg body weight. Histopathological examinations were carried out after 24 hours and after 1, 4 and 14 weeks. In addition, the BALF was analysed and the lung tissue was examined by immunohistochemistry. The histopathological examination found transient, but severe inflammation with infiltrates of neutrophilic granulocytes and, in the further course, granuloma formation. The mRNA and protein concentrations of numerous proinflammatory mediators such as IL-1β, IL-6, IL-8, TNF-α, MCP-1 and MIP-2 were increased in the lung tissue. The mRNA levels and protein concentrations had returned to the levels of the controls after 1 week (TNF-α) and after 4 weeks (IL-1β, IL-6, IL-8, MCP-1 and MIP-2) (Cho et al. 2007). The study does not provide an exact description of the silica used.

Fine and ultrafine colloidal amorphous silica particles with mean diameters of 230 nm and 14 nm, respectively, were administered to female ICR mice in a single dose of 3 mg per animal. The mice were examined 30 minutes and 2, 6, 12 and 24 hours after exposure. Bronchoalveolar degeneration and necrosis as well as neutrophilic inflammation in alveoli with particle-laden macrophages were found in both treatment groups at the histopathological examination. In addition, the ultrafine particles induced alveolar haemorrhage and severe necrosis after only 30 minutes. The findings show that ultrafine particles cause more severe inflammatory responses and damage in the lung tissues than fine particles (Kaewamatawong et al. 2005).

The same group of authors carried out another study that investigated ultrafine particles in male ICR mice at doses of 0, 0.3, 3, 10, 30 or 100 µg per animal. After 3 days, the BALF was analysed, histological changes were assessed and the body weights were determined. Moderate to severe inflammation and damage to the lung tissues were found in the 2 high dose groups. To evaluate the response over time, mice were exposed to 30 µg and examined over a period of up to 30 days. All effects were reversible (Kaewamatawong et al. 2006).

Ultrafine particles of amorphous silica with a particle size of 14 nm were administered once to groups of 5 male A/J mice by intratracheal instillation in doses of 0, 2, 10 or 50 mg/kg body weight. The animals were examined 1 day and 1, 4 or 14 weeks after exposure. Gomori’s trichrome staining revealed severe alveolar epithelial thickening and pulmonary fibrosis one week after exposure. For the most part, however, these effects had returned to the normal range after 4 weeks. The mRNA and protein levels of the cytokines IL-4, IL-10, IL-13, interferon-γ (IFN-γ) and matrix metalloproteinases (MMP-2, MMP-9, MMP-20) and matrix metalloproteinase inhibitor TIMP-1 were increased with statistical significance 1 day and 1 week after exposure. The mRNA and protein levels had returned to the control levels 4 and 14 weeks after exposure, respectively; those of IFN-γ and MMP-2 were the only exception. An exact characterization of the silica used in the study was not provided (Choi et al. 2008).

Fumed amorphous silica nanoparticles (AEROSIL^®^ 200) with a mean diameter of 12 nm were administered to ICR mice in a single dose of 1 mg/kg body weight. The animals were examined 1, 7, 14 and 28 days after exposure. Body weight gains were reduced with statistical significance after 1 day and after 7 days. Proinflammatory cytokines, cytotoxic T cells, NK cells, NKT cells and the profibrotic cytokine transforming growth factor-β (TGF-β) were increased. Micro-granulomatous changes were detected at the histopathological examination 7 and 14 days after exposure (Park et al. 2011).

### 5.2 Subacute, subchronic and chronic toxicity

#### 5.2.1 Inhalation

In a short-term study, 24 CD rats were exposed to the aerosol of the amorphous silica Zeofree 80 in concentrations of 0, 10 or 100 mg/m^3^ for 6 hours a day over a period of 3 days. A transient pulmonary inflammatory response was observed at concentrations of 10 mg/m^3^ and above that was still noticeable up to 1 day after the end of exposure, but had subsided by day 8 of the observation period (Warheit et al. 1995).

In a study investigating inhalation toxicity, male and female Wistar rats were exposed nose-only to 3 different forms of SAS in concentrations of 0, 1, 5 or 25 mg/m^3^ for 6 hours a day on 5 days. The test substances were agglomerates of precipitated silica (Zeosil 45), silica gel (Syloid) and fumed silica (Cab-O-Sil M5). Crystalline silica (quartz dust) was used as the positive control in a concentration of 25 mg/m^3^. Necropsy was performed 1 day or 1 or 3 months after the end of exposure. The authors reported only marginal differences between the SAS that were observed only on the first day after the end of exposure. No treatment-related effects were detected at 1 mg/m^3^. At concentrations of 5 mg/m^3^ and above, slight histopathological changes in the lungs were observed, such as increases in the lung weights and tracheobronchial lymph node weights, intra-alveolar accumulation of macrophages and granulocytes and bronchial/bronchiolar hypertrophy. Cytotoxicity, the biomarkers alkaline phosphatase (ALP), LDH, superoxide dismutase and the TNF-α levels in the BALF were likewise increased. For the most part, all effects had returned to the normal range after an observation period of 1 to 3 months (Arts et al. 2007).

In a subacute inhalation study, rats were exposed nose-only to colloidal SAS (Ludox) in concentrations of 0, 10, 50 or 150 mg/m^3^ over a period of 2 or 4 weeks. After exposure for 4 weeks, lung burdens of 489 µg/lung (10 mg/m^3^ group), 2418 µg/lung (50 mg/m^3^ group) and 7378 µg/lung (150 mg/m^3^ group) were determined. Exposure to colloidal SAS (Ludox) in a concentration of 150 mg/m^3^ caused inflammation in the lungs that was accompanied by increased protein levels, increased LDH and ALP activities in the BALF as well as reduced macrophage phagocytosis. Exposure to 50 mg/m^3^ caused an inflammatory response in the lungs that was characterized by an increased neutrophil count. Increased proliferation of pulmonary epithelial cells (labelling index increased after 2 weeks from 0.6% in the control group to 1.8% in the 150 mg/m^3^ group) was observed in the groups exposed to the 2 high concentrations both after exposure for 2 and 4 weeks. All parameters had returned to the control levels after a recovery period of 3 months (IARC 1997; Warheit et al. 1991).

In a subacute inhalation study, male SD rats were exposed nose-only to synthetic amorphous silica nanoparticles in concentrations of 0, 0.407 ± 0.066, 1.439 ± 0.177 or 5.386 ± 0.729 mg/m^3^ for 6 hours a day, on 5 days a week, for 4 weeks. The silica aerosols were specially made from tetraethyl orthosilicate using a nanoparticle generator. The generated particles were characterized by transmission electron microscopy (TEM) and energy-dispersive X-ray spectroscopy. The particle sizes were reported as 53 ± 2.56 nm for the low concentration group, 76 ± 1.53 nm for the medium concentration group and 79 ± 1.52 nm for the high concentration group. Exposure was followed by recovery phases of 1, 7 or 28 days. Five weeks after the beginning of the exposure, the body weights in the high concentration group were reduced with statistical significance in comparison with the values determined in the control group. No statistically significant changes were observed in the body weights of any other group or at any other time point. The erythrocyte counts and the haemoglobin concentrations were increased with statistical significance in all exposure groups on the day after the end of exposure. The histopathological examinations did not detect any significant differences from the values of the control group at any time point. Furthermore, the inflammatory parameters in the BALF were not increased (Shin et al. 2017).

Male F344 rats were exposed via whole-body inhalation to fumed silica (type NM-203) at concentrations of 0 or 50.4 ± 19.0 mg/m^3^ for 6 hours per day, on 5 days per week, over a period of 13 weeks. At the end of the 13-week exposure period, the mean lung burden was 882.7 ± 83.1 µg/lung. The total number of cells, the protein levels and the LDH and glucuronidase activities determined in the BALF were increased with statistical significance in comparison with the values found in the controls. The fraction of neutrophilic leukocytes in the total number of cells increased from 0.26% (controls) to 55% (NM-203), while the fraction of alveolar macrophages decreased from 98% (controls) to 43% (NM-203). These values had returned to the control levels within 3 months (Johnston et al. 2000).

In a 90-day inhalation study carried out according to OECD Test Guideline 413, 10 male and 10 female Wistar rats (albino) per concentration group were exposed via the nose to the synthetic amorphous silica HDK^®^ SKS 300 for 6 hours per day, on 5 days per week. The target concentrations of 0, 0.5, 2.0 and 10 mg/m^3^ were found to have been reached on average with measured values of 0, 0.51 ± 0.06, 2.05 ± 0.015 and 10.01 ± 0.47 mg/m^3^, respectively. The MMAD of the test substances was 2.80 ± 0.33 µm, 6.62 ± 0.28 µm and 4.47 ± 0.41 µm, respectively, in the low, medium and high concentration groups. Exposure was followed by a 13-week recovery period. On the first day without exposure, the activities of ALP in the medium concentration group, aspartate aminotransferase in the medium and high concentration groups and alanine aminotransferase (ALT) in the high concentration group were increased with statistical significance in the males. In the males of the low and high concentration groups, there was a statistically significant, but reversible decrease in sodium levels. A statistically significant increase in the absolute and relative weights of the lungs and tracheobronchial lymph nodes was determined in the medium and high concentration groups. These findings were still noticeable in the females at the end of the recovery period. A gross-pathological examination at the end of exposure found red discoloration of the lungs with white areas in 2 females of the high concentration group. The results of the histopathological examination are shown in Table 1. The histological examination at the end of exposure found an accumulation of alveolar macrophages and interstitial inflammation with cell infiltrates in all animals of the medium and high concentration groups. In addition, bronchoalveolar epithelial hyperplasia was observed in 10 of 10 males and in 9 of 10 females of the high concentration group and in 5 of 10 males and 5 of 10 females of the medium concentration group.

**Tab.1:** Histopathological findings in male and female rats in the 90-day inhalation study with HDK^®^ SKS 300, not including the findings at 0.5 mg/m^3^ (ECHA 2021)

**Findings**	**Severity grade**	**Concentration [mg/m^3^]**											
		**End of exposure**						**Recovery period**					
		**♂**			**♀**			**♂**			**♀**		
		** 0**	** 2**	**10**	** 0**	** 2**	**10**	** 0**	** 2**	**10**	** 0**	** 2**	**10**
**Lungs**													
Number of animals		**10**	**10**	**10**	**10**	**10**	**10**	**10**	** 5**	**10**	**10**	** 5**	**10**
Bronchoalveolar hyperplasia	very slight	1	5	0	1	5	7	0	1	3	0	0	0
	mild	0	0	10	0	0	2	0	0	2	0	0	1
Accumulation of alveolar macrophages	very slight	0	10	0	0	10	2	0	0	0	0	0	4
	mild	0	0	10	0	0	6	0	0	0	0	0	0
	moderate	0	0	0	0	0	0	0	0	0	0	0	0
Interstitial macrophages and lymphocyte infiltration	very slight	0	8	0	0	2	2	0	1	7	0	2	6
	mild	0	2	9	0	8	8	0	0	3	0	0	0
	moderate	0	0	1	0	0	0	0	0	0	0	0	0
**Mediastinal lymph nodes**													
Number of animals		**10**	**10**	**10**	**10**	**10**	**10**	**10**	** 4**	**10**	**10**	** 5**	**10**
Increased sinus histiocytosis	very slight	0	0	1	0	0	0	0	1	0	0	1	1
	mild	0	0	4	0	0	5	0	0	0	0	0	2
	moderate	0	0	3	0	0	2	0	0	3	0	0	3
Macrophage aggregation	very slight	0	6	1	0	6	0	0	3	1	0	3	1
	mild	0	2	3	0	0	3	0	0	4	0	0	4
	moderate	0	0	5	0	0	6	0	0	4	0	0	1

The changes observed in the group that was exposed to the low concentration of 0.5 mg/m^3^ were not described in detail. The changes corresponded in incidence and severity to those found in the control group. As a result, they are not considered exposure-related. Overall, the effects observed after the 13-week recovery period were less severe in incidence and severity, but not completely reversible (no other details; ECHA 2021). A copy of the original study is not available.

Another 90-day inhalation study was carried out according to OECD Test Guideline 413. Groups of 55 male Wistar rats (WU) were exposed nose-only to synthetic amorphous silica NM-200 for 6 hours per day on 5 days per week. On average, the concentrations reached the targets of 0, 1, 2.5 or 5 mg/m^3^ almost exactly (100%–104%). The test substance NM-200 is a precipitated silica that has the CAS number 112926-00-8 and a specific surface area (BET) of 160 m^2^/g. The MMAD was 2.16 ± 0.09 µm, 2.94 ± 0.20 µm and 3.12 ± 0.06 µm, respectively, in the low, medium and high concentration groups. Exposure was followed by a 13-week recovery period. A clinico-biochemical finding was a statistically significant increase in the number of polymorphonuclear neutrophils. This finding was considered exposure-related and was present across all concentration groups. In the medium and high concentration groups, the lymphocyte counts, LDH and β-glucuronidase activities and total protein levels were increased with statistical significance and the macrophages were reduced. In addition, a statistically significant increase in the number of polymorphonuclear neutrophils was observed in the low concentration group. All findings were reversible and had returned to the control levels 3 months after the end of exposure. A statistically significant increase in the cytokine CINC-1 concentration was observed on day 14, but not on the first day after the end of exposure; TNF-α and interleukin-6 remained unchanged. The absolute (5 mg/m^3^ group) and relative (2.5 and 5 mg/m^3^ groups) lung weights were increased with statistical significance. The absolute lung weights remained increased with statistical significance in the 2.5 and 5 mg/m^3^ groups at the end of the recovery period. The histopathological examination detected statistically significant findings of multifocal mucous goblet cell hyperplasia and epithelial hyaline droplets in addition to multifocal infiltration of inflammatory cells in the nasal cavity even at the lowest concentration tested (1 mg/m^3^) and above. Alveolar infiltration of granulocytes in the lungs was increased with statistical significance in the medium and high concentration groups. The findings of interstitial macrophage infiltration and granulocyte infiltration in the lungs were statistically significant in the high concentration group. Unlike the findings in the lungs, the effects in the nasal cavity were still noticeable, but less severe, at the end of the recovery period. On the basis of these data, REACH registrants have established a LOAEC (lowest observed adverse effect concentration) of 1 mg/m^3^ for the effects in the nasal cavity (no other details; ECHA 2021). The original study report is not available.

In a 90-day inhalation study, groups of 70 male and 70 female Wistar rats were exposed to different types of synthetic amorphous silica for 6 hours per day on 5 days per week and observed for up to a year after the end of exposure. The test substances were aerosols of Aerosil^®^ 200 in target concentrations of 0, 1, 6 and 30 mg/m^3^ (actual concentrations 0, 1.3 ± 0.1, 5.9 ± 0.2 and 31 ± 0.9 mg/m^3^) and of Aerosil^®^ R 974 and Sipernat^®^ 22 S, each in a target concentration of 30 mg/m^3^ (actual concentrations 34.7 ± 0.7 and 34.9 ± 0.5 mg/m^3^, respectively). To establish a comparison, quartz dust was likewise tested in a concentration of 60 mg/m^3^ (actual concentration 58.5 ± 0.7 mg/m^3^). An MMAD was not given for the tested particles. The examinations were carried out directly after the end of the exposure period and at the end of the recovery periods of 13, 26, 39 and 52 weeks. Directly after the end of exposure, the number of neutrophilic granulocytes found in all groups was higher than the number found in the controls. However, the increase was statistically significant only in the group exposed to Aerosil^®^ 200 in a concentration of 30 mg/m^3^ and in the quartz dust group. Thirteen weeks after the end of exposure, this increase was no longer detectable in the Aerosil® 200 group but persisted in the quartz group. The increase in neutrophilic granulocytes progressed in parallel with changes in the lungs. The alterations induced by synthetic amorphous silica were most pronounced at the end of the exposure period and subsided at varying rates over the course of 1 year following exposure. Aerosil^®^ 200 caused the most severe and Sipernat^®^ 22 S the least severe effects. All test substances led to the accumulation of alveolar macrophages, polymorphonuclear leukocytes, cellular debris and intra-alveolar granular material as well as alveolar bronchiolization. The increased accumulation of alveolar macrophages remained statistically significant at the end of the exposure period in all exposed groups and at all tested concentrations. This effect subsided over the course of the recovery period, but was increased with statistical significance up to week 39 after exposure to Aerosil^®^ R 974 and week 52 in the group exposed to an Aerosil^®^ 200 concentration of 30 mg/m^3^. Alveolar bronchiolization was evident to a statistically significant degree up to week 26 of the recovery period particularly in the male rats of the Aerosil^®^ 200 group that was exposed to a concentration of 30 mg/m^3^. At the end of the exposure period, a statistically significant increase was observed for the accumulation of cellular debris in the 2 groups that were exposed to higher concentrations of Aerosil^®^ 200 (5.9 ± 0.2 and 31 ± 0.9 mg/m^3^) and for the number of intra-alveolar polymorphonuclear leukocytes in all groups exposed to Aerosil^®^ 200. However, both effects had returned to their initial values by the end of the 52-week recovery period. At the end of exposure, a statistically significant increase in septal cellularity was observed in all animals exposed to Aerosil^®^ 200 and in the females exposed to Aerosil^®^ R 974. The values gradually returned to normal levels, depending on the concentration. The increase in septal cellularity remained statistically significant in the females and males that were exposed to Aerosil^®^ 200 concentrations of 30 mg/m^3^ even 52 weeks after exposure. Focal interstitial fibrosis, seen as amorphous eosinophilic, collagen-containing thickening of the septa, was first observed 13 weeks after exposure in nearly all exposed groups except for the males that were exposed to an Aerosil^®^ 200 concentration of 1 mg/m^3^ and the females that were exposed to Aerosil^®^ R 974, Sipernat^®^ 22 S and quartz dust. The changes in the Aerosil^®^ R 974 and Sipernat^® ^22 S groups subsided completely over the further course of the recovery period. However, the changes became more marked in the group exposed to 30 mg/m^3^ of Aerosil^®^ 200 and in the quartz dust group (Reuzel et al. 1991).

About 30 years later, the test material that was still available from the study of Reuzel et al. (1991) (males; max. 10 animals per group) was re-stained, examined and re-evaluated. The re-evaluation was performed by a pathology working group (PWG) (Hardisty 2016). The working group found a series of degenerative and inflammatory changes in the samples of all test groups and control groups. Table 2 and Table 3 provide an overview of the most important changes and their severity in addition to data for significance. According to these findings, daily inhalation exposure to Aerosil^®^ 200 (1.3–31 mg/m^3^), Aerosil^®^ R 974 (34.7 mg/m^3^) and Sipernat^®^ 22 S (34.9 mg/m^3^) did not cause irreversible damage in the lungs during the observation period. Fibrosis was not observed either at the end of exposure or after the 13-week recovery period. Minimal fibrosis (not statistically significant) was found at later examination time points in 2 animals of the group exposed to the highest Aerosil^®^ 200 concentration. After a recovery period of 52 weeks, minimal focal fibrosis was detected in 1 animal of the control group and in 1 animal each of the groups exposed to the low and medium concentrations of Aerosil^®^ 200 and in 3 animals of the group exposed to the high Aerosil^®^ 200 concentration. No fibrosis was noticeable in the groups exposed to Aerosil^®^ R 974 and Sipernat^®^ 22 S. Other than several reactive changes in the form of the occurrence and accumulation of alveolar macrophages, the findings in the group exposed to the high concentration of Aerosil^®^ 200 did not differ with statistical significance from those found in the control animals (Hardisty 2016; Weber et al. 2018).

**Tab.2:** Incidences and severity grades (in parentheses) of the most important changes in the lungs of male rats, modified according to Weber et al. (2018)

**Findings**	**TP**	**Concentration [mg/m^3^]**			
		**0**	**1.3**	**5.9**	**31**
Pneumocytes type II alveolar hyperplasia (= normal architecture, but alveoli are lined predominantly by type II cells)	0	0/9	6/10* (1.5)^a)^	9/10* (2.0)	8/9* (2.8)
	13	0/5	0/5	3/5 (1.3)	4/5* (4.2)
	52	1/10 (1.0)	0/10	0/10	4/10 (1.0)
Alveolar and bronchiolar epithelial hyperplasia (= focal or multifocal areas of increased cellularity)	0	0/9	5/10* (1.2)	5/10* (1.4)	3/9 (2.0)
	13	0/5	1/5 (1.0)	4/5* (1.0)	3/5 (1.7)
	52	1/10 (3.0)	3/10 (1.3)	0/10	3/10 (1.0)
Granulomatous inflammation	0	0/9	5/10* (2.2)	7/10* (2.0)	6/9* (1.7)
	13	0/5	0/5	1/5 (1.0)	5/5* (1.8)
	52	0/10	0/10	0/10	2/10 (1.0)
Granulomas, alveolar/bronchiolar junctions (= focal accumulation of histiocytes/macrophages at alveolar/bronchiolar junctions)	0	0/9	3/10 (2.3)	6/10* (1.7)	7/9* (1.3)
	13	0/5	0/5	1/5 (1.0)	4/5* (1.0)
	52	0/10	0/10	0/10	0/10
Interstitial inflammation	0	3/9 (1.3)	6/10 (1.0)	5/10 (1.4)	8/9* (1.1)
	13	1/5 (1.0)	0/5	2/5 (1.0)	3/5 (1.3)
	52	2/10 (1.0)	2/10 (1.0)	2/10 (1.0)	4/10 (1.0)
Granulomas with cholesterol clefts (= focal formation of granulomas with clefts)	0	0/9	0/10	0/10	2/9 (2.0)
	13	0/5	0/5	2/5 (1.0)	5/5* (1.6)
	52	0/10	0/10	1/10 (1.0)	3/10 (1.0)
Foreign material in alveoli	0	0/9	1/10 (1.0)	10/10* (2.0)	9/9* (2.4)
	13	0/5	0/5	0/5	1/5 (1.0)
	52	0/10	0/10	0/10	0/10
Alveolar macrophages	0	2/9 (1.0)	8/10* (2.8)	10/10* (3.5)	9/9* (4.3)
	13	1/5 (1.0)	4/5 (1.0)	5/5* (1.8)	5/5* (2.0)
	52	4/10 (1.0)	5/10 (1.2)	5/10 (1.0)	5/10 (1.0)
Fibrogenesis	0	0/9	0/10	0/10	0/9
	13	0/5	0/5	0/5	2/5 (1.0)
	52	0/10	0/10	0/10	2/10 (2.0)

^*^ p < 0.05

TP: time point of examination; TP 0: after exposure for 13 weeks, TP 13: after recovery for 13 weeks, TP 52: after recovery for 52 weeks

^a)^ for information about the severity of the change see Table 3

**Tab. 3:** Diagnostic criteria and grade of change, modified according to Weber et al. (2018)

**Finding**	**Description**	**Grade**
Alveolar pneumocyte type II hyperplasia	Normal architecture, but alveoli are lined predominantly by type II cells	1: < 3% of alveolar / bronchiolar junctions affected
		2: < 6% of alveolar / bronchiolar junctions affected
		3: < 15% of alveolar / bronchiolar junctions affected
		4: < 30% of alveolar / bronchiolar junctions affected
		5: > 30% of alveolar / bronchiolar junctions affected
Alveolar/bronchiolar epithelial hyperplasia	Focal or multifocal areas of increased cellularity, whereby the bronchiolar-alveolar architecture is still detectable. Epithelial cells are dominant and mostly single layered	1: one area (focus) minimally affected
		2: several areas (foci) minimally affected
		3: all areas (foci) minimally affected
		4: several areas (foci) moderately affected
		5: several areas (foci) severely enlarged
Granuloma, alveolar/bronchiolar	Focal accumulation of histiocytes/macrophages at alveolar/bronchiolar junctions	1: < 5 of alveoli affected
		2: < 10 of alveoli affected
		3: < 20 of alveoli affected
		4: < 30 of alveoli affected
		5: > 30 of alveoli affected
Granuloma with cholesterol clefts	Focal granuloma formation with clefts	1: < 5 of alveoli affected
		2: < 10 of alveoli affected
		3: < 20 of alveoli affected
		4: < 50 of alveoli affected
		5: > 50 of alveoli affected
Foreign material in alveoli	Residuals of inhaled test substance that was deposited within the alveolar lumina. The material appeared as small fine granular particles, often accompanied by cellular remnants of macrophages	1: minimal, in 1 or 2 alveoli
		2: up to 40% of alveoli affected
		3: approximately 40%–60% of alveoli affected
		4: approximately 80%–90% of alveoli affected
		5: all alveoli affected
Alveolar macrophages	Isolated reactive macrophages containing inhaled material in cytoplasm within alveolar lumina (alveolar histiocytosis)	1: < 10% of alveoli affected
		2: < 25% of alveoli affected
		3: < 50% of alveoli affected
		4: < 75% of alveoli affected
		5: > 75% of alveoli affected
Fibrogenesis	Increases in septal or interstitial thickness resulting from oedema or inflammation without substantial fibre cross-linking. In the present study associated with minimal inflammatory infiltration that is considered to be fully reversible	1: one area (focus) minimally affected
		2: several areas (foci) minimally affected
		3: all areas (foci) minimally affected
		4: several areas (foci) moderately affected
		5: several areas (foci) severely enlarged

In a 90-day inhalation study carried out by Fraunhofer ITEM (2020) according to OECD Test Guideline 413, groups of 225 male and 225 female Wistar rats [Crl:WI (Han)] were exposed nose-only to 2 different types of synthetic amorphous silica for 6 hours a day on 5 days a week. SAS1-high BET (SAS1) and SAS2-low BET (SAS2) aerosols were tested in target concentrations of 0, 0.5, 1, 2.5 or 5 mg/m^3^. The actual concentrations were within the range of 97% to 110%. The test substance SAS1 is a form of synthetic amorphous silica with a specific surface area of 400 m^2^/g, a primary particle size of 5 to 10 nm and an MMAD of 2.08 to 3.04 µm. SAS2 is a form of synthetic amorphous silica with a specific surface area of 40 to 50 m^2^/g, a primary particle size of 30 to 40 nm and an MMAD of 1.30 to 2.20 µm. The solubility of the test substances at 20 °C was determined using 2 different methods (ICP-OES and photometry). The solubility of SAS1 was determined to be 232.6 ± 6.8 mg/l (photometry) and 226.5 ± 10.5 mg/l (ICP-OES) and that of SAS2 to be 127.7 ± 7.3 mg/l (photometry) and 122.6 ± 11.7 mg/l (ICP-OES). SAS1 is more soluble because it has a higher fraction of OH groups, particularly terminal OH groups, on its surface. Thermogravimetric analysis was used to determine the different numbers of OH groups on the surface. The animals were examined 1 day (+1), 90 days (13 weeks), 180 days (25 weeks) or 360 days (52 weeks) after the end of exposure. A group of 10 males and 10 females was examined at the +1 time point, while the examination at all other time points was carried out with groups of 5 males and 5 females. Five animals died during the study period; however, a causal relationship with the exposure could not be established. Systemic toxicity and sex-specific differences were not observed. The body weight gains and the food and water consumption of the test animals did not differ with statistical significance from the values in the control animals. In the groups exposed to SAS1, a statistically significant increase in absolute and relative lung weights was observed in the females of the high concentration group (5 mg/m^3^); this effect was no longer noticeable 13 weeks after exposure. Analysis of the BALF revealed a statistically significant increase in polymorphonuclear neutrophilic granulocytes (PMN) and a statistically significant decrease in macrophages in the groups exposed to medium (2.5 mg/m^3^) and high (5 mg/m^3^) concentrations of SAS1 irrespective of the sex (Table 4 and 5). The animals in both concentration groups recovered within 13 weeks. In the groups exposed to SAS2, a statistically significant increase in the absolute and relative lung weights was observed in both sexes at concentrations of 1 mg/m^3^ and above. Evidence of this effect was still noticeable in the high concentration group 13 weeks after the end of exposure. In the SAS2 groups, the PMN in the BALF was increased with statistical significance and there was a statistically significant decrease in macrophages in both sexes and in all concentration groups. The animals recovered fully: the group exposed to 0.5 mg/m^3^ within 13 weeks, the group exposed to 1 mg/m^3^ within 25 weeks and the groups exposed to concentrations of 2.5 mg/m^3^ and above within 52 weeks after the end of exposure. IL-8 was increased with statistical significance in both sexes and in all concentration groups after exposure to both SAS1 and SAS2.

**Tab. 4:** Cytological parameters (mean values ± SD; n = 10) in the bronchoalveolar lavage fluid after 90-day inhalation exposure of male Wistar rats to SAS1 and SAS2 (Fraunhofer ITEM 2020)

**SAS concentration**	**Leukocyte concentration [cells/ml]**	**Macrophages [%]**	**PMN [%]**	**Lymphocytes [%]**
0 mg/m^3^	115 813 ± 38 494	98.6 ± 1.0	1.1 ± 0.9	0.3 ± 0.4
SAS1				
0.5 mg/m^3^	83 813 ± 22 021	94.1 ± 8.8	5.6 ± 8.8	0.3 ± 0.3
1 mg/m^3^	107 438 ± 33 005	89.4 ± 6.7	9.7 ± 5.8	1.0 ± 1.6
2.5 mg/m^3^	194 813 ± 79 578	77.8 ± 10.6*	21.5 ± 10.5*	0.7 ± 0.7
5 mg/m^3^	315 250 ± 233 570	51.0 ± 11.3*	48.3 ± 11.6*	0.7 ± 0.6
SAS2				
0.5 mg/m^3^	309 813 ± 193 092	73.2 ± 12.9*	25.9 ± 13.0*	0.9 ± 0.9
1 mg/m^3^	830 625 ± 398 652	58.0 ± 9.6*	41.6 ± 9.8*	0.5 ± 0.5
2.5 mg/m^3^	1 482 000 ± 949 026	51.7 ± 7.5*	48.2 ± 7.6*	0.2 ± 0.3
5 mg/m^3^	1 458 000 ± 590 279	46.4 ± 5.5*	52.9 ± 5.6*	0.7 ± 0.8

^*^ p < 0.001, Dunnett’s test (see text)

PMN: polymorphonuclear neutrophilic granulocytes

**Tab. 5:** Cytological parameters (mean values ± SD; n = 10) in the bronchoalveolar lavage fluid after 90-day inhalation exposure of female Wistar rats to SAS1 and SAS2 (Fraunhofer ITEM 2020)

**SAS concentration**	**Leukocyte concentration [cells/ml]**	**Macrophages [%]**	**PMN [%]**	**Lymphocytes [%]**
0 mg/m^3^	100 125 ± 36 235	99.1 ± 1.0	0.5 ± 1.0	0.4 ± 0.4
SAS1				
0.5 mg/m^3^	101 875 ± 26 321	99.5 ± 0.4	0.3 ± 0.2	0.2 ± 0.2
1 mg/m^3^	90 250 ± 20 188	96.5 ± 3.0	3.2 ± 3.0	0.3 ± 0.2
2.5 mg/m^3^	163 625 ± 73 594	70.0 ± 8.5*	29.0 ± 8.9*	1.0 ± 0.8
5 mg/m^3^	198 650 ± 87 015	60.8 ± 10.0*	38.4 ± 10.1*	0.8 ± 0.4
SAS2				
0.5 mg/m^3^	180 313 ± 71 973	77.7 ± 10.5*	21.3 ± 10.8*	1.0 ± 0.5
1 mg/m^3^	373 750 ± 109 795	59.2 ± 6.7*	40.4 ± 7.0*	0.5 ± 0.5
2.5 mg/m^3^	759 500 ± 484 018	53.9 ± 7.4*	45.4 ± 7.3*	0.7 ± 0.6
5 mg/m^3^	1 322 000 ± 558 774	49.5 ± 10.6*	49.5 ± 10.1*	1.0 ± 0.7

^*^ p < 0.001, Dunnett’s test (see text)

PMN: polymorphonuclear neutrophilic granulocytes

The histological examination of the nasal cavity revealed a proliferation of goblet cells in section level 1 at all SAS1 concentrations, but without concentration dependency (5, 6, 1, 3 animals in the 4 SAS1 groups; controls: 0). The severity did not increase up to 2.5 mg/m^3^. The proliferation of goblet cells was observed in section level 2 and in the nasopharyngeal duct only at the highest exposure concentration. An increase in the incidence and severity of hyaline inclusions in the olfactory mucosa was found in the exposed animals over the course of the study. In addition, Chitinase-positive crystals were detected in the olfactory mucosa (section levels 2–4) up to week 26 after the end of exposure, particularly in the samples taken from the animals exposed to SAS1.

The gross-pathological examination revealed discoloration in the lungs of the animals exposed to SAS2 in concentrations of 1 mg/m^3^ and above. The associated inflammatory changes increased in incidence and/or severity depending on the concentration. The histopathological findings in the lungs are described in detail in Table 6 (SAS1) and Table 7 (SAS2). Perivascular infiltration was increased after exposure to SAS1 concentrations of 1 mg/m^3^ and above and after exposure to all concentrations of SAS2. Alveolar macrophages and macrophage agglomeration increased with the concentration after exposure to both test substances, whereby the effects were more pronounced in the SAS2 groups. However, it is important to note that, unlike the findings reported for the males, the females of the control group had a high incidence (8/10) of increased alveolar macrophage counts. However, the Commission does not regard these effects as adverse. Macrophage type II hyperplasia was observed in the animals exposed to SAS1 in concentrations of 1 mg/m^3^ and above (only 1 animal affected) and at a higher incidence in those exposed to concentrations of 2.5 mg/m^3^ and above. Again, a higher overall incidence was observed in the animals of the SAS2 concentration groups and the effect was already noticeable in the 0.5 mg/m^3^ group. Low-grade interstitial inflammation was observed at concentrations of 0.5 mg/m^3^ and above; however, no concentration–effect relationship was evident. Low-grade interstitial inflammation was observed at all SAS2 concentrations and at higher incidences in the SAS2 groups than were found in the SAS1 groups. In the groups treated with SAS1, granulomas were observed at bronchoalveolar junctions in the males exposed to concentrations of 1 mg/m^3^ and above and in the females of the high concentration group. In the groups exposed to SAS2, this effect was observed in both males and females at all concentrations tested. In individual animals of the SAS1 groups, these changes were associated with low-grade bronchoalveolar hyperplasia; the incidence in the SAS2 groups was considerably higher. Fibrogenesis was detected in 2 males and 1 female in the control groups. In the animals exposed to SAS1 concentrations of 1 mg/m^3^ and above, a higher incidence of fibrogenesis was found than in the control animals. The incidence was considerably higher in the animals of the SAS2 concentration groups and was already noticeable in the 0.5 mg/m^3^ group. Macrophage agglomeration in the bronchus-associated lymphoid tissue (BALT) was found in 1 male in the SAS1 group exposed to 1 mg/m^3^. The number of animals affected increased slightly at SAS1 concentrations of 2.5 mg/m^3^ and above. Neither hyperplasia nor granulomatous inflammation of the BALT was observed in the animals of the SAS1 groups. In comparison, hyperplasia, granulomatous inflammation and macrophage agglomeration were observed in the BALT of animals exposed to SAS2 at concentrations as low as 0.5 mg/m^3^ and above (Table 6 and 7).

The histopathological findings in the lungs of the animals exposed to SAS1 were fully reversible after a recovery period of 13 weeks, except for slight agglomeration of alveolar macrophages in the groups exposed to concentrations of 2.5 and 5 mg/m^3^. In comparison, the animals exposed to SAS2 in concentrations of 1 mg/m^3^ and above required a recovery period of more than 52 weeks. Non-reversible interstitial fibrosis of the lungs or an increase in the collagen content were not observed after exposure to SAS1 or SAS2. The 2 forms of SAS did not differ in terms of the qualitative characteristics of pathogenicity and the pathological findings (Fraunhofer ITEM 2020).

**Tab. 6:** Histological findings induced by SAS1 in the lungs after 90-day inhalation exposure of Wistar rats (Fraunhofer ITEM 2020)

**Findings**	**SAS1 concentration**									
	**0 mg/m^3^**		**0.5 mg/m^3^**		**1.0 mg/m^3^**		**2.5 mg/m^3^**		**5.0 mg/m^3^**	
	**♂**	**♀**	**♂**	**♀**	**♂**	**♀**	**♂**	**♀**	**♂**	**♀**
Number of animals	**9**	**10**	**10**	**10**	**10**	**10**	**10**	**10**	**10**	**10**
Perivascular infiltration	1/1.0^a)^	1/1.0	1/1.0	0	4/1.3	3/1.0	4/1.0	2/1.0	1/1.0	2/1.0
Alveolar macrophages	1/1.0	8/1.0	8/1.0	7/1.0	10/1.3	10/1.0	10/1.7	10/1.7	10/1.6	10/1.5
Macrophage agglomeration	0	0	2/1.0	4/1.0	6/1.0	6/1.0	8/1.1	9/1.1	9/1.0	6/1.0
Macrophage II hyperplasia	0	0	0	0	1/1.0	0	6/1.0	3/1.0	6/1.2	2/1.0
Inflammation, interstitial	0	0	0	1/1.0	2/1.0	0	5/1.0	2/1.0	1/1.0	1/1.0
Granulomas with cholesterol clefts	0	0	0	0	0	0	0	0	0	0
Granulomas (junctions)	0	0	0	0	2/1.0	0	4/1.0	0	9/1.0	9/1.0
Alveolar/bronchiolar hyperplasia	0	0	0	0	1/1.0	0	4/1.0	0	1/1.0	0
BALT hyperplasia	0	0	0	0	0	0	0	0	0	0
BALT macrophage agglomeration	0	0	0	0	1/1.0	0	2/1.0	1/1.0	5/1.2	1/1.0
BALT granulomatous inflammation	0	0	0	0	0	0	0	0	0	0
MT: fibrogenesis	2/1.0	1/1.0	0	1/1.0	3/1.0	1/1.0	6/1.0	5/1.0	8/1.0	7/1.0

^a)^ incidence/severity; no data for statistical significance

BALT: bronchus-associated lymphoid tissue; MT: Masson’s trichrome stain

**Tab. 7:** Histological findings induced by SAS2 in the lungs after 90-day inhalation exposure of Wistar rats (Fraunhofer ITEM 2020)

**Findings**	**SAS2 concentration**									
	**0 mg/m^3^**		**0.5 mg/m^3^**		**1.0 mg/m^3^**		**2.5 mg/m^3^**		**5.0 mg/m^3^**	
	**♂**	**♀**	**♂**	**♀**	**♂**	**♀**	**♂**	**♀**	**♂**	**♀**
Number of animals	**9**	**10**	**10**	**10**	**10**	**10**	**10**	**10**	**10**	**10**
Perivascular infiltration	1/1.0^a)^	1/1.0	6/1.2	1/1.0	6/1.7	4/1.0	4/1.0	6/1.0	6/1.3	7/1.1
Alveolar macrophages	1/1.0	8/1.0	10/1.8	10/1.9	10/2.4	10/2.3	10/2.8	10/2.9	10/2.9	10/3.0
Macrophage agglomeration	0	0	10/1.5	10/1.3	8/1.3	9/1.3	9/1.4	8/1.4	9/1.6	10/1.8
Macrophage II hyperplasia	0	0	7/1.0	7/1.1	8/1.5	9/1.6	10/1.6	10/1.1	10/1.8	9/1.4
Inflammation, interstitial	0	0	10/1.4	10/1.1	10/1.8	7/1.6	7/1.0	9/1.7	10/1.4	10/1.7
Granulomas with cholesterol clefts	0	0	0	0	0	0	1/1.0	0	0	0
Granulomas (junctions)	0	0	9/1.2	5/1.6	6/1.7	6/1.5	9/1.2	6/1.5	9/1.2	5/1.2
Alveolar/bronchiolar hyperplasia	0	0	7/1.1	1/1.0	3/1.0	1/1.0	0	1/1.0	1/1.0	1/1.0
BALT hyperplasia	0	0	1/1.0	0	0	0	0	1/2.0	0	0
BALT macrophage agglomeration	0	0	4/1.0	4/1.0	6/1.5	1/1.0	2/1.5	4/1.8	6/1.8	6/1.7
BALT granulomatous inflammation	0	0	0	1/2.0	0	0	0	5/1.8	1/1.0	2/1.5
MT: fibrogenesis	2/1.0	1/1.0	7/1.0	9/1.0	6/1.0	7/1.0	7/1.0	9/1.0	10/1.0	7/1.0

^a)^ incidence/severity; no data for statistical significance

BALT: bronchus-associated lymphoid tissue; MT: Masson’s trichrome stain

The gross-pathological examination carried out at the end of the exposure period revealed enlarged lymph nodes in the animals exposed to SAS1 concentrations of 1 mg/m^3^ and above and in all animals exposed to SAS2. Granulomas in the mediastinal and tracheobronchial lymph nodes were detected in the animals exposed to SAS1 concentrations of 1 mg/m^3^ and above. Granulomatous inflammation was found only in the male animals of the groups exposed to SAS1 concentrations of 2.5 mg/m^3^ and above. Lymphoid hyperplasia was observed in all concentration groups exposed to either SAS1 or SAS2. However, the effect was not dependent on the concentration. Granulomas and granulomatous inflammation were found in the animals exposed to SAS2 at all concentrations, even the lowest concentration. Additionally, fibrogenesis or fibrosis was observed in the animals exposed to SAS2. Overall, the effects were more marked in the SAS2 groups than in the SAS1 groups and occurred at a higher incidence (Table 8 and 9) (Fraunhofer ITEM 2020).

**Tab. 8:** Histological findings induced by SAS1 in the mediastinal and tracheobronchial lymph nodes observed on the day following the end of exposure (+1) (Fraunhofer ITEM 2020)

**Findings**	**SAS1 concentration**									
	**0 mg/m^3^**		**0.5 mg/m^3^**		**1.0 mg/m^3^**		**2.5 mg/m^3^**		**5.0 mg/m^3^**	
	**♂**	**♀**	**♂**	**♀**	**♂**	**♀**	**♂**	**♀**	**♂**	**♀**
**Mediastinal lymph nodes**										
Number of animals	**7**	**2**	**6**	**7**	**6**	**4**	**6**	**8**	**10**	**8**
Granulomas	0	0	0	0	2/2.0^a)^	2/1.0	5/2.0	6/1.3	0	4/2.3
Granulomatous inflammation	0	0	0	0	0	0	0	0	0	0
Lymphoid hyperplasia	0	0	0	0	2/2.0	1/2.0	4/2.0	3/2.0	0	0
MT: fibrogenesis	0	0	0	0	0	0	0	0	0	0
MT: fibrosis	0	0	0	0	0	0	0	0	0	0
**Tracheobronchial lymph nodes**										
Number of animals	**10**	**9**	**10**	**6**	**8**	**8**	**7**	**8**	**10**	**9**
Granulomas	0	0	0	0	2/1.5	3/1.7	4/2.0	6/1.8	10/2.5	9/2.7
Granulomatous inflammation	0	0	0	0	0	0	4/2.0	0	1/1.0	0
Lymphoid hyperplasia	0	0	1/2.0	1/1.0	0	1/2.0	2/1.5	2/1.5	1/2.0	3/1.0
MT: fibrogenesis	0	0	0	0	0	0	0	0	0	0
MT: fibrosis	0	0	0	0	0	0	0	0	0	0

^a)^ incidence/severity; no data for significance

MT: Masson’s trichrome stain

**Tab. 9:** Histological findings induced by SAS2 in the mediastinal and tracheobronchial lymph nodes observed on the day following the end of exposure (+1) (Fraunhofer ITEM 2020)

**Findings**	**SAS2 concentration**									
	**0 mg/m^3^**		**0.5 mg/m^3^**		**1.0 mg/m^3^**		**2.5 mg/m^3^**		**5.0 mg/m^3^**	
	**♂**	**♀**	**♂**	**♀**	**♂**	**♀**	**♂**	**♀**	**♂**	**♀**
**Mediastinal lymph nodes**										
Number of animals	**7**	**2**	**9**	**9**	**10**	**10**	**10**	**10**	**9**	**8**
Granulomatous inflammation	0	0	2/1.5^a)^	4/2.5	8/1.1	9/2.2	9/1.9	10/2.0	1/2.0	5/2.0
Lymphoid hyperplasia	0	0	8/1.8	6/1.5	10/2.0	9/2.2	10/2.2	10/2.2	0	4/2.0
MT: fibrogenesis	0	0	0	2/1.0	9/1.0	5/1.0	8/1.0	6/1.0	0	3/1.0
MT: fibrosis	0	0	0	1/1.0	0	1/1.0	3/1.3	6/1.2	0	2/1.0
**Tracheobronchial lymph nodes**										
Number of animals	**10**	**9**	**10**	**9**	**9**	**7**	**10**	**6**	**10**	**9**
Granulomas	0	0	4/3.0	6/2.5	2/2.0	5/2.2	3/3.0	4/2.8	10/3.8	9/3.6
Granulomatous inflammation	0	0	6/1.8	2/2.0	4/2.3	2/2.5	6/3.0	3/2.0	7/1.9	8/2.0
Lymphoid hyperplasia	0	0	5/2.0	0	2/2.0	1/1.0	5/2.0	0	10/1.8	6/2.2
MT: fibrogenesis	0	0	1/1.0	2/1.0	0	3/1.0	2/1.0	2/1.0	2/1.0	1/1.0
MT: fibrosis	0	0	0	0	2/1.0	1/2.0	6/1.0	0	9/1.3	8/1.3

^a)^ incidence/severity; no data for significance

MT: Masson’s trichrome stain

**Summary**: The percentage of PMN and the number of macrophages in the BALF were increased with statistical significance at SAS1 concentrations of 2.5 mg/m^3^ and above and at SAS2 concentrations of 0.5 mg/m^3^ and above. Although the results of Dunnett’s test do not show statistical significance for PMN at a concentration of 1 mg/m^3^, the test is not suitable for evaluating these data because the variance is not homogenous. A post-hoc analysis using the Games-Howell test found that the percentage of PMN was increased with statistical significance (p < 0.05) even at a SAS1 concentration of 1 mg/m^3^. In the histological examination, the first concentration-dependent effects were detected in the SAS1 groups at 1 mg/m^3^, but these were not statistically significant. The histological effects detected at a SAS2 concentration of 0.5 mg/m^3^ were much more severe. This applied to interstitial inflammation, the increased incidence of granulomas, alveolar and bronchiolar hyperplasia and fibrogenesis. Also, more severe inflammation developed in the lymph nodes after exposure to SAS2 than after exposure to SAS1; this effect was already noticeable at the lowest concentration of 0.5 mg/m^3^. For the histopathological findings detected on the first day after the end of exposure (+1), the authors of the study derived a NOAEC of 1 mg/m^3^ for SAS1-high BET and a LOAEC of 0.5 mg/m^3^ for SAS2-low BET. The Commission established a LOAEC of 1 mg/m^3^ for SAS1 based on the interstitial inflammation and granulomas that occurred in 2 of 10 males in each case, and the 10-fold higher fraction of PMN found in the males at this concentration. A NOAEC of 0.5 mg/m^3^ was determined for SAS1.

An inhalation study investigated the effects induced by 3 different types of synthetic amorphous silica (silica gel, precipitated silica and fumed silica) in groups of 10 male Cynomolgus monkeys, 80 male Sprague Dawley rats and 20 male Hartley guinea pigs at a concentration of 15 mg/m^3^. The animals were exposed for 5.5 to 6 hours a day, on 5 days a week, over a period of up to 18 months. The animals were examined histopathologically; the rats after 3, 6 and 12 months, the guinea pigs after 12 months and the monkeys after 13 months (silica gel and fumed silica) and 18 months (precipitated silica). The amount of amorphous silica found in the lungs of the rats was only about 10% of that found in the lungs of the monkeys. Particle-loaded macrophages and cell infiltrates were found in the monkey lungs; these effects were much less pronounced in the rats and guinea pigs. Alveolar granulomas were detected in 6 of the 9 monkeys that were exposed to fumed silica; in 5% to 50% of the cases, the granulomas contained collagen. No or almost no collagen was found in the granulomas from 3 monkeys and in all samples taken from the pulmonary interstitium of the monkeys. Several of the examined rats had interstitial fibrosis; however, this effect was likewise detected in a number of the control animals. Unlike the samples collected from the monkeys, the tissue sections from the rat lungs were not examined for reticulin and collagen using special dyes. Mica (“KAlSiO_3_”) and kaolin (“AlSiO_3_”) were detected in varying amounts in several monkeys from each group (including the controls). However, according to the authors, this finding had no influence on the effects that were observed because, on the one hand, the lungs in which the minerals were found did not contain larger amounts of reticulin or collagen and, on the other hand, no mica or kaolin was found in the group with the highest collagen levels (fumed silica). In addition, no collagen was found in the lungs of the control group animals that contained mica. How the animals were exposed to mica and kaolin was not described (Groth et al. 1981). Based on the particle size distribution given by the authors, at most 42% of the particle mass at a concentration of 15 mg/m^3^ had an aerodynamic diameter below 3.3 µm. OECD Test Guideline 412 for inhalation studies in rats prescribes that the MMAD should not be higher than 3 µm. In this study, the respirable particle mass/m^3^ for rats would have been at most 6.3 mg/m^3^. In comparison, about 80% (12 mg/m^3^) of the particles would have reached the pulmonary alveoli in the monkeys.

#### 5.2.2 Oral administration

In a 28-day study, 4 different types of amorphous silica, some with surface functionalization, were administered by gavage to male and female Wistar rats (Crl:WI(Han)). The study was performed as a limit test with doses of 0 or 1000 mg/kg body weight and day. All 4 types had a mean particle size of 15 nm. The study was carried out according to OECD Test Guideline 407. None of the types of silica tested induced adverse, substance-related effects (Buesen et al. 2014).

No substance-induced symptoms were observed in rats given different types of synthetic amorphous silica (NM-200, UB19157, Aerosil R 504) for 4 weeks in daily gavage doses of up to 1000 mg/kg body weight and day. Substance-related symptoms were likewise not found in a feeding study with fumed silica (8157-7) in concentrations of up to 2250 mg/kg feed (equivalent to 188 mg/kg body weight) that was carried out under the same conditions as described above (ECHA 2021).

Other oral studies are included in the REACH registration dossier. None of these studies found evidence of substance-specific toxicity (ECHA 2021).

In a study, 2 types of fumed synthetic amorphous silica (SAS and NM-202) were administered in the feed to groups of 5 male SD rats for 28 days in doses of 0, 100, 1000 or 2500 mg/kg body weight and day (SAS) and in doses of 0, 100, 500 or 1000 mg/kg body weight and day (NM-202). The animals in the high dose groups were exposed to SAS or NM-202 for 84 days. The SAS particles were between 10 and 25 nm in size, had a specific surface area of 200 m^2^/g and a purity ≥ 99.9%. The NM-202 particles had a mean size of 7 nm, a specific surface area of 380 m^2^/g and a purity of ≥ 99.8%. After exposure for 84 days, deposits of only SAS were found in the spleen. The incidence of liver fibrosis was increased in the high dose groups after exposure to both forms of silica, but reached statistical significance only in the animals exposed to NM-202. This effect was accompanied by a moderate, but statistically significant increase in the expression of fibrosis-related genes in the liver (van der Zande et al. 2014).

A communication questioned the results published by van der Zande et al. (2014) and provided a detailed description of the shortcomings of the study, which included the method used to characterize the material, the study design, the definitions and the determination of the end points as well as the statistical evaluation (Morfeld et al. 2017).

#### 5.2.3 Dermal application

In a study published in 1958, Cab-O-Sil in 0.5% methyl cellulose was applied to the intact or abraded skin of 2 male and 2 female rabbits per group in doses of 5000 or 10 000 mg/kg body weight and day for 18 hours a day, on 5 days a week, for 3 weeks. The body weights remained unchanged. According to the abstract, the histopathological examination did not reveal any substance-induced adverse effects other than irritation at the application site (ECHA 2021).

A study that was carried out according to OECD Test Guideline 411 with a subchronic duration of exposure examined the toxicity of colloidal silica particles with a diameter of 20 nm after dermal application to 10 male and 10 female Sprague Dawley rats per group. Doses of 0, 500, 1000 or 2000 mg/kg body weight and day were applied to the skin of the animals for 90 days. Gauze soaked with a SiO_2_-nanoparticle solution was fixed to a hairless area on the back of the animals for 6 hours every day. Over the course of the study and during the observation of an additional 5 male and 5 female animals of the dose groups exposed to 0 and 2000 mg/kg body weight, effects such as decreased water intake were observed and reversible alopecia occurred in 1 female animal (days 46–49) of the 500 mg/kg group. SiO_2_ did not induce irritation or histological changes on the skin. Several pathological changes were observed in the organs, but these were not statistically significant. However, the described effects were not dependent on the dose and were therefore evaluated as coincidental. Changes to several haematological parameters (increased lymphocyte count, reduced neutrophil count) and clinical parameters (reduced creatinine kinase, increased ALT) were observed; these were likewise evaluated as coincidental because they were not accompanied by changes to the clinical appearance or by histopathological findings (Ryu et al. 2014).

#### 5.2.4 Intraperitoneal injection

The studies are summarised in Section 5.7.

#### 5.2.5 Intratracheal instillation

The studies are summarized in Section 5.7.

#### 5.2.6 Intravenous injection

Male and female BALB/c mice and female C57BL/6 mice were exposed to fine silica particles 70 nm in size at doses of 0, 30 or 40 mg/kg body weight and day. The particles were injected intravenously twice a week over a period of 4 weeks. The exact type of silica used was not described. The test materials were designated merely as SP70, SP70-N and SP70-C. These designations correspond to silica particles with a diameter of 70 nm that have not undergone surface modification or have been modified with one amino or carboxyl group, respectively. Liver damage in the form of fibrosis was observed in the exposed animals, but surface-modified materials induced less severe damage than unmodified SP70. Additionally, a significant, dose-dependent increase in ALT was found in the groups exposed to SP70-N and SP70-C (Fruijtier-Pölloth 2012; Isoda et al. 2011).

### 5.3 Local effects on skin and mucous membranes

#### 5.3.1 Skin

A total of 19 studies were carried out with different forms of amorphous silica (BET 45–700 m^2^/g) to investigate skin irritation in rabbits. The test substances were applied both to intact skin and to abraded skin for a period of up to 24 hours. The studies found at most minimal irritation of the skin, which was attributed to the hygroscopic properties of the material and its mechanical effects (ECHA 2021).

#### 5.3.2 Eyes

A total of 24 studies were carried out with different forms of amorphous silica (BET 45–700 m^2^/g) to investigate eye irritation in rabbits. The amorphous silica induced effects in the eyes ranging from no irritation to conjunctivitis with complete recovery within 48 hours. No statistically significant damage or irreversible irritation was induced in the eyes. The mild irritation in the eyes was attributed to the hygroscopic properties of the material and its mechanical effects (ECHA 2021).

### 5.4 Allergenic effects

In a modified local lymph node assay (LLNA) that was not carried out according to OECD test guidelines, a formulation containing 1 mg of colloidal or mesoporous silica nanoparticles in ethanol was applied to 5 female BALB/c mice per group on both ears from days 1 to 3. The control animals were exposed to a mixture of acetone and olive oil (4:1). The ear thickness was measured on days 1 to 3 and on days 4 and 6. On day 6, the animals were administered ^3^H-thymidine by injection. The auricular lymph nodes were excised 5 hours later. The authors suggested that the results for colloidal silica were positive because the ear thickness increased (very) little from days 2 to 6 (Lee et al. 2011). Because lymphocyte proliferation was not increased on day 6, the results are considered negative. The registration dossier for synthetic amorphous silica includes other studies that investigated sensitization using similar substances, for example those assigned the CAS numbers 126877-03-0, 67762-90-7 and 68909-20-6 (ECHA 2021). These likewise yielded negative test results, but were not included in the evaluation because of the chemical modifications.

### 5.5 Reproductive and developmental toxicity

#### 5.5.1 Fertility

In a 2-generation study carried out according to OECD Test Guideline 416, synthetic amorphous silica (NM-200, precipitated) was given by gavage to groups of 28 male and 28 female Wistar rats in doses of 0, 100, 300 or 1000 mg/kg body weight and day. The mean hydrodynamic diameter of the amorphous silica particles (dispersion in 0.5% aqueous hydroxypropyl methylcellulose) was between 1076 to 1664 nm in the 10 g/l sample and between 876 and 1216 nm in the 30 g/l sample. The particles in the 100 g/l sample seem to have been the smallest in diameter at 409 to 703 nm, but the particles formed a sediment and agglomerated because of the high concentration of particles in the sample. No toxicity or adverse effects on fertility were observed in the parent animals up to the high dose. A NOAEL (no observed adverse effect level) of 1000 mg/kg body weight and day was established for toxicity and for the fertility of the parent animals (Wolterbeek et al. 2015).

No adverse effects on fertility or perinatal toxicity were found in a generation study with administration of synthetic amorphous silica (fumed) via the feed to Wistar rats in a dose of 500 mg/kg body weight and day. The study was not carried out according to an OECD test guideline and had some limitations. Only one dose was tested in the study and a small number of animals was used (ECETOC 2006; EFSA Panel on Food Additives and Nutrient Sources added to Food (ANS) et al. 2018; Fruijtier-Pölloth 2016; Lewinson et al. 1994).

#### 5.5.2 Developmental toxicity

In a prenatal developmental toxicity study carried out according to OECD Test Guideline 414, synthetic amorphous silica nanoparticles were given to Wistar rats in gavage doses of 0, 100, 300 or 1000 mg/kg body weight and day from days 6 to 19 of gestation. The primary particles (NM-200) consisted of precipitated amorphous silica with a SiO_2_ fraction of 96.5%. The particles were 10 to 25 nm in size. No maternal toxicity was observed up to the high dose. The foetal weights and the number of foetuses did not differ from the values determined in the control group. A substance-related increase in the incidence of malformations and variations in the foetuses was not found. The NOAEL for developmental toxicity and maternal toxicity was therefore 1000 mg/kg body weight and day, the highest dose tested (Hofmann et al. 2015).

In the 2-generation study with Wistar rats described in Section 5.5.1, no adverse effects on litter parameters in the F1 and F2 generations or on sexual maturation and the oestrus cycle in the F1 generation were found in the offspring up to the highest dose tested of 1000 mg/kg body weight and day. A NOAEL for maternal toxicity of 1000 mg/kg body weight and day was derived (Wolterbeek et al. 2015) in addition to a NOAEL for perinatal toxicity of 1000 mg/kg body weight and day.

An oral study investigating synthetic amorphous silica (gel) found no adverse effects on fertility and no prenatal developmental toxicity in Wistar rats, CD-1 mice, Dutch belted rabbits and hamsters up to the highest doses tested of 1350 mg/kg body weight and day (rats, mice) and 1600 mg/kg body weight and day (rabbits, hamsters) (ECETOC 2006; EFSA Panel on Food Additives and Nutrient Sources added to Food (ANS) et al. 2018; Fruijtier-Pölloth 2016). The study has some limitations. The documentation and the statistical analysis are not adequate and the investigations were not carried out according to the OECD test guidelines valid today. Therefore, the study was not included in the evaluation.

### 5.6 Genotoxicity

#### 5.6.1 In vitro

In a number of different bacterial mutagenicity tests, silica did not induce any mutagenic activity in the Salmonella typhimurium strains TA98, TA100, TA1535, TA1537, TA1538 and in Escherichia coli either with or without the addition of a metabolic activation system. Furthermore, amorphous silica did not cause chromosomal aberrations in various mammalian cell lines. Silica was not mutagenic in the TK^+/–^ mutation test with L5178Y mouse lymphoma cells or in the HPRT gene mutation test with a cell line derived from Chinese hamster ovary (CHO cells) (ECHA 2021).

Nanoparticles of two fumed, one precipitated and 2 colloidal types of amorphous silica were examined by comet assay to investigate the cause of DNA strand breaks. In the assay, 3T3-L1 fibroblasts were incubated with 0, 4 or 40 µg/ml of silica for 3, 6 and 24 hours. The incidence of DNA strand breaks was not increased with statistical significance at either of the 2 silica concentrations (Barnes et al. 2008).

In the comet assay, amorphous silica with mean particle sizes of 15 or 55 nm did not induce relevant DNA damage in V79 cells at the non-cytotoxic concentrations of 100 µg/ml and above and of 300 µg/ml and above, respectively. In a test for the induction of DNA strand breaks using an alkaline unwinding technique, the incidence of DNA strand breaks was increased with statistical significance only in the cells treated with the 15 nm-sized particles in a concentration of 100 µg/ml. Oxidative base damage was not evident when measured using Fpg-sensitive sites (Maser et al. 2015).

The amorphous silica nanoparticles SiNP12 (12 nm), SiNP5-15 (5–15 nm), SiNP10-20 (10–20 nm) and SiP2 (2 µm) were investigated for cytotoxicity and genotoxicity in lung epithelial cells (FE1) derived from Muta^TM^ mice. The hydrodynamic diameter in the medium was determined by DLS (dynamic light scattering) and the primary particle size was determined by transmission electron microscopy (TEM). The primary particle sizes corresponded to the manufacturer’s information. The TEM results show that some of the amorphous silica SiP2 particles were 1 µm in size. The samples were analysed for chemical impurities by ICP-MS (inductively coupled plasma mass spectrometry). Traces of sodium, aluminium, calcium, titanium, iron and zirconium were found. The cytotoxicity was determined by trypan blue staining 24 hours after exposure to 0, 12.5, 25 or 50 µg/ml. In a range-finding study, concentrations of 100 µg/ml and above led to total cell death. Cell viability decreased with the increase in concentration and was between 53% and 95% at the low concentration. The smaller particles induced stronger cytotoxic effects. Seven days after the cells were treated for 24 hours with particles in a concentration of 12.5 µg/ml, the number of colonies formed (a measure of the proliferation capacity of the cells) was decreased with statistical significance only in the cells treated with SiNP12. Reactive oxygen species (ROS) were generated, with the highest number induced by SiP2 (SiP2 > SiNP12 > SiNP10–20 > SiNP5–15); the course was biphasic. The simultaneously determined glutathione levels showed an opposite trend to the increases or decreases of the ROS. Only one concentration (12.5 µg/ml) was examined in the micronucleus test and in the lacZ mutation test, as higher concentrations induced cell death. The micronuclei count was increased with statistical significance in cells treated with the particles SiNP12 (1.62-fold), SiNP5-15 (1.74-fold) and SiNP10-20 (1.5-fold). SiP2 did not induce micronuclei. According to the authors, the increase in micronuclei correlated with an increase in the surface area of the SiNP species. The different types of SiNP did not induce mutations in the lacZ gene (Decan et al. 2016). However, only 1000 cells per experiment were analysed in the micronucleus test instead of the 2000 cells required by the test guideline. In addition, only one concentration was tested.

The incidence of micronuclei was not increased after human peripheral lymphocytes were incubated for 24 hours with amorphous silica with a mean particle size of 15 nm (Levasil^®^ 200/40) and 55 nm (Levasil^®^ 50/50) in concentrations of 31.6 to 1000 µg/ml. With the 15 nm-sized particles, precipitates were formed at the highest concentration tested of 1000 µg/ml (Downs et al. 2012).

Amorphous silica with mean particle sizes of 15 nm (Levasil^®^ 200/40) and 55 nm (Levasil^®^ 50/50) was investigated for its genotoxic potential in Caco-2 cells. In the cytokinesis-block micronucleus assay carried out according to OECD Test Guideline 487, the number of micronuclei in binucleated cells increased with the concentration only in the cells treated with 15 nm-sized particles. This effect already occurred at 16 µg/ml, a concentration that is not yet cytotoxic. H2AX phosphorylation was increased only in the cells treated with 15 nm-sized particles. As apoptosis occurred concurrently, the DNA damage, probably double-strand breaks, is regarded as an indirect effect. The cytotoxicity induced by the 15 nm-sized particles was statistically significant at 32 µg/ml. No cytotoxicity was observed in the cells treated with 55 nm-sized particles up to a concentration of 120 µg/ml (Tarantini et al. 2015 b).

#### 5.6.2 In vivo

After groups of 5 male Wistar rats were given a single intratracheal instillation of amorphous silica particles in an amount of 360 µg per animal, genotoxic effects in the lungs or bone marrow were not induced in the 72 hours that followed, either by the 15 nm-sized or by the 55 nm-sized particles. A comet assay and a micronucleus test were performed. The ratio of polychromatic to normochromatic erythrocytes (PCE/NCE) remained unchanged. According to the authors, the negative results of the micronucleus test indicate low systemic availability, making it doubtful that the bone marrow was reached (Maser et al. 2015).

Two types of precipitated amorphous silica (NM-200 and NM-201) and two of fumed amorphous silica (NM-202 and NM-203) were investigated for potential genotoxic effects. The materials were obtained from the EU Joint Research Centre Nanomaterials Repository and were well characterized. The substances were administered to groups of 5 male Sprague Dawley rats by three intratracheal injections of 0, 3, 6 or 12 mg/kg body weight (cumulative doses 0, 9, 18 or 36 mg/kg body weight) 48, 24 and 3 hours before a tissue sample was taken. In addition, groups of 6 male Sprague Dawley rats were given 3 intravenous injections containing particles of the type NM-203 in doses of 0, 5, 10 or 20 mg/kg body weight (cumulative doses 0, 15, 30 or 60 mg/kg body weight). The genotoxic effects in the erythrocytes were determined by a micronucleus test carried out according to OECD Test Guideline 474. Additionally, a modified comet assay was performed with BAL, lung, blood, spleen, kidney, liver and bone marrow cells. The micronuclei in the bone marrow were increased with statistical significance only in the group that received an intravenous injection of the high dose of NM-203. The PCE/NCE ratio was no different from that of the control groups, but the incidence of mortality in this dose group was very high with 3 of 6 animals. The data obtained after intravenous injection were not included in the evaluation for the same reasons as apply for the study of Downs et al. (2012) (see below). When compared with the findings obtained with the positive controls (methyl methanesulfonate and *N*-ethyl-*N*-nitrosourea), the silica nanoparticles did not induce genotoxic effects after intratracheal administration. However, it is doubtful that the particles reached the bone marrow because of their low systemic availability (Guichard et al. 2015).

Another amorphous silica study investigated alkaline (pH ~10) aqueous dispersions of colloidal silica with mean particle sizes of 15 nm (Levasil^®^ 200/40) and 55 nm (Levasil^®^ 50/50). The particles were diluted in phosphate buffer prior to intravenous injection and adjusted to a pH of 7.5. The amorphous silica was administered to male Wistar rats (n = 6–8, 15-nm particles 50 mg/kg body weight n = 4, positive controls and control group in the second experiment n = 3) in doses of 0, 25 or 50 mg/kg body weight (15 nm-sized particles) or in doses of 0, 25, 50 or 125 mg/kg body weight (55 nm-sized particles) by 3 successive intravenous injections into the tail vein 48, 24 and 4 hours prior to sacrifice. The highest dose in this study was chosen based on the maximum tolerable dose (MTD) determined in an earlier range-finding study (n = 2, up to 100 mg/kg body weight for 15 nm-sized particles and 200 mg/kg body weight for 55 nm-sized particles). The genotoxic potential in the blood and tissues from the liver and lungs was examined by comet assay and a micronucleus test was carried out with circulating reticulocytes. The 15 nm-sized particles induced a statistically significant increase in DNA damage in the liver tissue (Dunnett’s Multiple Comparison Test) only at the high dose. However, this effect was not observed when the assay was repeated. A significant increase in DNA damage was not found in the lung tissue or in the white blood cells. The 15 nm and 55 nm-sized particles led to an increase in the percentage of reticulocytes containing micronuclei (1.5-fold to 2.1-fold) only in the range of the MTD (Downs et al. 2012). Intravenous injections represent the worst-case scenario. The findings obtained after intravenous administration were not confirmed by the studies of Maser et al. (2015) or Guichard et al. (2015) after intratracheal exposure or by Tarantini et al. (2015 a) after oral exposure. The effects induced after intravenous administration were observed only in the range of the MTD and could not be verified in a second experiment. The standard deviations are probably very large because of the small number of animals, which calls the statistical significance of the findings into question. For this reason, the study has not been included in the evaluation.

Male Sprague Dawley rats were given 2 types of precipitated amorphous silica (NM-200 and NM-201) and 2 of fumed amorphous silica (NM-202 and NM-203) on 3 consecutive days in gavage doses of 0, 5, 10 or 20 mg/kg body weight. The comet assay (pH 8.0) and the Fpg-modified comet assay did not reveal DNA damage in the blood, bone marrow, liver, spleen, kidneys, duodenum and large intestines. Furthermore, the number of micronuclei in the bone marrow was not increased. However, it is unclear whether the bone marrow was reached because the PCE to NCE ratio did not differ from that of the control animals. In the large intestines, the number of micronuclei was increased with statistical significance by NM-202 and NM-203 at the low dose of 5 mg/kg body weight. A dose–response relationship was not observed. However, the authors believe that uptake may be increased because of lower agglomeration at the low dose. No effects on plasma malondialdehyde levels, a marker for lipid peroxidation, were observed (Tarantini et al. 2015 a).

Male F344 rats were exposed by inhalation to fumed silica of the type NM-203 in concentrations of 0 or 50.4 ± 19.0 mg/m^3^ for 6 hours a day, on 5 days a week, for 13 weeks. The lung burden was 882 µg per lung. The HPRT test (ex-vivo study) did not reveal an increase in mutation frequency in the alveolar epithelial cells of the lungs (Johnston et al. 2000).

#### 5.6.3 Summary

Mutations were not observed in bacterial genotoxicity tests. Amorphous silica did not induce chromosomal aberrations in mammalian cells. No mutations were detected in the TK^+/–^ mutation test, in the lacZ mutation test or in the HPRT gene mutation test. In vitro, amorphous silica nanoparticles led to the formation of micronuclei and DNA strand breaks in the comet assay and in an alkaline unwinding assay. These effects were not observed when larger particles were tested. The dispersibility and agglomeration of the particles are dependent on the culture medium and on the surface reactivity. This may be the reason why smaller particles cause more severe effects in vitro. The available data do not provide evidence of the induction of mutations or of clastogenic or aneugenic effects by amorphous silica in vivo via routes of exposure that are relevant to the workplace.

### 5.7 Carcinogenicity

In a study, fumed silica made hydrophobic with dimethyldichlorosilane was given to male and female Sprague Dawley rats with the feed in a concentration of 100 mg/kg for 24 months. Effects were observed in very isolated cases, including a benign tumour of the mamma in a male rat. As the incidence was within the range of the historical controls for the rat strain, the tumour was regarded as coincidental (Lewinson et al. 1994).

Four groups of 40 male and 40 female B6C3F1 mice and Fischer rats, respectively, were given feed containing 0, 1.25%, 2.5% or 5% micronized silica (Syloid 244; SiO_2_ × n H_2_O) over their entire lifetime. The amount of silica ingested each day in the dose groups was equivalent to 1.2 to 1.88, 2.52 to 6.61 and 5.27 to 7.49 g/kg body weight, respectively, in the male mice and 1.02 to 2.57, 1.82 to 4.9 and 3.95 to 13.31 g/kg body weight, respectively, in the female mice. Male rats ingested doses of 0.4 to 0.71, 0.83 to 1.46 and 1.76 to 3.0 g/kg body weight, respectively, and the females received 0.4 to 0.75, 0.83 to 1.45 and 1.78 to 3.21 g/kg body weight, respectively. At the end of week 93 or 103, respectively, the average total uptake was 38.45, 79.78 and 160.23 g per male mouse, 37.02, 72.46 and 157.59 g per female mouse, 143.46, 179.55 and 581.18 g per male rat and 107.25, 205.02 and 435.33 g per female rat. Malignant lymphomas had formed in the haematopoietic organs of the mice, particularly in the medium dose group. However, these were evaluated as coincidental because a dose dependency could not be established. No treatment-related tumours were found in rats (IARC 1997; Takizawa et al. 1988).

In a study in male and female Syrian hamsters, fine particles of a mixture of amorphous and crystalline silica were administered intratracheally in a dose of 3 mg per animal at weekly intervals over a period of 20 weeks. The animals were observed for the rest of their lives. The exact particle sizes were not specified, and the silica was not characterized in detail. No treatment-related tumours were found (IARC 1997).

After 10-week intratracheal administration of 3 mg of synthetic amorphous silica per animal (total dose 30 mg) followed by the observation of the animals for 2 years, the incidence of lung tumours in female Wistar rats was not increased with statistical significance (Pott and Roller 2005; Roller 2008).

Amorphous silica in the form of a particle suspension was repeatedly administered to female Wistar rats by intratracheal instillation over a period of 2 years. The aim was to generate and maintain chronic inflammation in the lungs similar to that produced by respirable biopersistent granular dusts without known specific carcinogenicity. However, the inflammation was not clearly attributable to the occurrence of biopersistent granular dusts in the lungs. As a result of the solubility of silica, the particles are eliminated more rapidly than quartz, leading on the one hand to the long-known, severe acute toxicity caused by highly dispersive amorphous silica and on the other hand to the regression of induced inflammatory responses and granulomas at the end of exposure. Pilot studies were carried out to investigate the elimination of amorphous SiO_2_ from the lungs of rats (Ernst et al. 2002, 2005). Two days after instilling 2 mg of amorphous silica, 18% of the silica was still present in the lungs; the half-life of these particles after instillation was 11 days. In the main study, 0.5 mg of fumed amorphous silica was given 30 times by instillation at intervals of 2 weeks to maintain a high level of cytotoxicity and inflammation over a period of about 15 months. The aim was to ascertain whether tumours would form at this level of cytotoxicity should this type of mechanism of action be the decisive cause. The particles had a mean diameter of 14 nm and a purity of 99.8%. The animals did not exhibit any adverse effects during the tests. Breathing problems developed shortly after administration, but were transient. After 9 months, body weight gains were reduced with statistical significance. The histopathological examination found multifocal, moderate to severe alveolar and interstitial accumulations of particle-laden macrophages in the lungs that were dose-dependent. Moderate to severe chronic granulomatous inflammation was detected in the lymph nodes of the lungs. A routine histology (6 sections/lung) detected lung tumours in 5 of 53 rats that had survived for at least a year (9.4%). An extensive histological examination of the lungs (60 sections/lung) was carried out in 31 rats that had survived for at least 2 years. Another 5 animals were thus found to have lung tumours, thereby increasing the number of animals with lung tumours from 5 (routine histology; 1 animal with a lung tumour died after 98 weeks and, as a result, did not undergo the second histological examination (60 sections/lung)) to a total of 10 of 53 (18.9%). No tumours were found in the lungs of the 55 rats of the control group, either at the time of the routine histology or during the extensive histology (60 sections/lung) that was carried out in 30 animals (survival: at least 2 years). A positive control group of 53 animals was treated with an intratracheal instillation of 3 mg of DQ 12 quartz. Only 7 of the animals of this group were examined histopathologically; these were all animals that had survived for at least 2 years. Lung tumours were documented in 6 of the 7 animals; however, 44 tumours (previously 17) were detected after increasing the number of sections. The histopathological examination revealed various types of tumours in all animals: bronchioalveolar carcinomas, bronchioalveolar adenomas, squamous cell carcinomas and cystic-keratinizing epitheliomas. The authors did not specify which tumour types were found in which of the groups treated with the different particles (Kolling et al. 2008). The results for amorphous silica are of only limited relevance because the 30 intratracheal instillations administered at intervals of 2 weeks induced a high level of ongoing acute cytotoxicity and inflammation. Furthermore, the study did not use different doses. On the one hand, this method of administration made it possible to achieve a more persistent lung burden with the soluble particles in spite of the solubility of amorphous SiO_2_. On the other hand, however, the lungs had a higher cytotoxic burden due to the dissolved SiO_2_. Overall, these results are not suitable as evidence that carcinogenic effects are induced in humans.

### 5.8 Other effects

Glycol-coated nanoparticles were used to study the allergy-promoting effects of colloidal amorphous silica on the airways. Initially, the authors administered silica (90 nm) intranasally in female BALB/c mice in doses of 0, 10, 100 or 400 µg/animal in combination with ovalbumin for sensitization. The animals were treated again with the allergen ovalbumin 14 and 15 days after sensitization. One day later, the animals were sacrificed and examined. Co-exposure to silica during sensitization gave rise to adjuvant-like effects that intensified the allergic reaction induced in the airways by ovalbumin in a dose-dependent manner. Ovalbumin-specific IgE-antibodies in the serum, eosinophilic granulocyte infiltration and mucosal cell metaplasia were increased in mice exposed to both substances. The gene and protein expression of Th2 and Th17 cytokines was likewise increased (Brandenberger et al. 2013).

To investigate the inflammation-promoting mechanisms of amorphous silica nanoparticles, RAW 264.7 cells, a mouse peritoneal macrophage cell line, were treated with particles that had a mean diameter of 12 nm. An increase in ROS was accompanied by a decrease in intracellular glutathione. In addition, a spectrophotometric analysis found increased nitric oxide levels. The authors assume that the ROS trigger reactions in the cells that promote inflammation. The authors did not provide an exact characterization of the silica (Park and Park 2009).

Aggregates of different sizes made up of precipitated amorphous silica nanoparticles with primary particle sizes of 14 to 23 nm were used to investigate the effects on cell metabolic activity, cell viability, glutathione content and IL-8 and IL-6 secretion in human bronchial epithelial cells (HBE), colon epithelial cells (Caco-2) and monocytic cells (THP-1) after exposure for 24 hours. The aggregates had a mean equivalent circle diameter of 100 to 2000 nm and mean hydrodynamic diameter of 264 nm to 12.5 µm. The authors used dynamic light scattering to demonstrate that the effects caused by the aggregated silica were generally less severe than those induced by non-aggregated nanoparticles (Murugadoss et al. 2020).

In another study investigating synthetic amorphous silica (equivalent to the types in Barnes et al. 2008) at concentrations of 0, 12.5, 25, 50 or 100 µg/ml, a statistically significant reduction in cell viability was observed after exposure of V79 cells to fumed silica at concentrations of 25 µg/ml and above and to precipitated SAS at concentrations as low as 12.5 µg/ml. However, these effects were induced only by nanoparticles that were about 20 nm in size. Furthermore, fumed and colloidal particles of this size induced DNA damage and apoptosis, but without producing intracellular ROS. Such effects were not observed with larger particles (50 nm). The authors concluded that toxicity was dependent more on the size of the particles than on the type of synthetic amorphous silica (fumed or precipitated) (Guichard et al. 2015).

## 6 Manifesto (MAK value/classification)

The critical effect is the occurrence of inflammatory responses in the lungs after inhalation exposure to synthetic amorphous silica. In general, it is not possible to make distinctions by particle size or surface composition because of the production processes used to produce the different types of synthetic amorphous silica, precipitated silica or fumed silica.

**MAK value. **A cross-sectional study by Taeger et al. (2016 b) cannot be used for the derivation of a MAK value due to methodological shortcomings. Fibrosis was not observed at any time point. The study demonstrated that the effects induced by amorphous silica differ qualitatively from those of crystalline forms (quartz).

In an animal study carried out by Reuzel et al. (1991), intra-alveolar infiltration of PMN and septal cellularity were increased with statistical significance equally in male and female rats in the low concentration group (1.3 mg/m^3^). An increase in alveolar bronchiolization was an additional finding detected at the next-higher concentration (5.9 mg/m^3^). This effect was observed only in the male rats and was not statistically significant.

An inhalation study carried out by Groth et al. (1981) investigated 3 types of amorphous silica in monkeys, rats and guinea pigs. The findings revealed a marked inter-species difference in the respirable particle concentrations. Based on the particle size distribution given by the authors, at most 42% of the particle mass at a concentration of 15 mg/m^3^ had an aerodynamic diameter below 3.3 µm. However, OECD test guidelines for inhalation studies in rats prescribe that the MMAD should not be greater than 3 µm. The respirable particle concentration for rats would therefore be at most 6.3 mg/m^3^. In monkeys, by contrast, agglomerates with higher aerodynamic diameters (about 80%) would be able to reach the lungs. Therefore, the respirable particle concentration in monkeys would be at most 12 mg/m^3^. The respirable particle concentration determined by Groth et al. (1981) for rats is thus equivalent to the middle concentration of Reuzel et al. (1991). The different respirable particle concentrations may explain why the rat lungs contained only about 10% of the amount of amorphous silica that was found in the monkey lungs. Alveolar granulomas were detected in 6 of the 9 monkeys; in 5% to 50% of the cases, the granulomas contained collagen. No collagen was found in the granulomas of 3 of the 9 monkeys. None of the animals had increased levels of collagen in the pulmonary interstitium. There was no evidence of fibrotic processes in the lungs of the monkeys. The interstitial fibrosis observed in rats, which were exposed to respirable silica at much lower levels than the monkeys, was detected also in several control animals. The available data are not sufficient to draw the conclusion that rats are less sensitive than monkeys.

The histopathological findings in the study of Reuzel et al. (1991) were reviewed by Weber et al. (2018) and re-evaluated by an expert committee (Pathology Working Group, PWG). All findings and grades are described in detail in Weber et al. (2018). Only the sections from the male rats were available for re-evaluation. After exposure of the rats by inhalation for 13 weeks, the incidence of pneumocyte type II hyperplasia, alveolar-bronchiolar hyperplasia and granulomatous inflammation was increased with statistical significance at the low concentration (1.3 mg/m^3^) and above. The severity of the granulomatous inflammation did not increase with the concentration. In addition to the changes that had already become noticeable at the low concentration, the formation of alveolar/bronchiolar granulomas reached statistical significance at the medium concentration (5.9 mg/m^3^). This finding (accumulation of histiocytes/macrophages) was already observed in the low concentration group, but the incidence was not statistically significant. The incidence and severity of the alveolar hyperplasia do not seem to be related to the concentration. Low-grade interstitial inflammation was statistically significant only at the high concentration (31 mg/m^3^). This finding was noticeable also at the lower concentrations and in the control animals (3/9), but was not statistically significant and its severity did not increase with the concentration. In this study, the finding “interstitial inflammation” is not classified as an adverse effect because it was observed also in the controls and no concentration dependency was established. The changes described by Weber et al. (2018) in the low and medium concentration groups are not regarded as clearly adverse mainly because of their type (regenerative), their low grade and the lack of an increase in severity and/or the only slight increase in severity. The data available are not suitable for drawing conclusions about possible effects after chronic exposure.

Report 02G18002 is a well-documented study that was carried out according to the state of the art in compliance with OECD Test Guideline 413 (Fraunhofer ITEM 2020). An increase in the absolute and relative lung weights can be evaluated as reaching the MTD. The MTD for SAS1 (BET 400 m^2^/g) is 5 mg/m^3^ and for SAS2 (BET 40–50 m^2^/g) 1 mg/m^3^. On the first day after the end of exposure (+1), PMN levels in the BALF were increased with statistical significance in both sexes at SAS2 concentrations as low as 0.5 mg/m^3^ and above. Increased IL-8 values correspond with the increase in PMN and are further markers of the treatment-induced inflammatory response in the lungs; the latter is supported by histological evidence. The histopathological findings in the lungs of the animals that were exposed to medium (1 mg/m^3^) and high (5 mg/m^3^) SAS2 concentrations required unusually long recovery periods of over 52 weeks. After a recovery period of 12 months, the interstitial inflammation was still noticeable in all but the lowest concentration group (0.5 mg/m^3^). The PMN fraction may not have been increased with statistical significance after exposure to a SAS1 concentration of 1 mg/m^3^, but the values in the males were almost 10 times as high as those of the control animals (Table 4), which was evaluated as adverse by the Commission. In this study, a NOAEC of 0.5 mg/m^3^ for SAS1-high BET and a LOAEC of 0.5 mg/m^3^ for SAS2-low BET were derived for the first day after the end of exposure (+1). The PMN data for SAS1 were used to estimate the NAEC (no adverse effect concentration) for SAS2: the PMN fraction at a SAS1 concentration of 2.5 mg/m^3^ was about the same as the fraction at a SAS2 concentration of 0.5 mg/m^3^. The corresponding NOAEC for SAS1 is 0.5 mg/m^3^. The same LOAEC/NOAEC ratio (5:1) is assumed for SAS2. As a result, a NAEC of 0.1 mg/m^3^ has been derived for SAS2. Using the MPPD model version 3.04 for particles with an MMAD of 2 µm and above (geometric standard deviation 2), it was calculated that the percentage of particles deposited in the tracheobronchial and pulmonary airways at a respiratory volume of about 20 l/min is only half that deposited under resting conditions at a volume of 7.5 l/min. This means that for relatively large particles in the respirable fraction, the mass deposited in the lower respiratory tract during breathing at rest and increased breathing is approximately the same. As the SAS at the workplace have a very large MMAD (Morfeld et al. 2014), the respirable fraction is probably made up of larger particles. Therefore, the higher respiratory volume of humans at the workplace in comparison with that of animals exposed under resting conditions in an experimental setting does not need to be taken into consideration.

The concentration of 0.5 mg/m^3^ has been established as the LOAEC for synthetic amorphous silica. After extrapolating the LOAEC to the NAEC (1:5, see above), subchronic to chronic exposure (1:2) and the data from animal studies to humans (1:2), a MAK value of 0.02 mg/m^3^ has been derived for the respirable fraction.

**Peak limitation. **The cumulative effects on the lungs are the critical effect of synthetic amorphous silica. The respirable fraction has therefore been assigned to Peak Limitation Category II. The different types of amorphous silica are cleared from the alveoli at different rates; fumed silica is eliminated from the lungs quite rapidly (Henschler 1991). However, no valid data are available to derive the elimination half-life for clearance from the lungs. As acute irritation in the respiratory tract was not reported after inhalation exposure and the histopathological changes in the lungs are long-term effects, an excursion factor of 8 has been established.

**Prenatal toxicity. **In a prenatal developmental toxicity study (Hofmann et al. 2015) and a 2-generation study (Wolterbeek et al. 2015) carried out in Wistar rats according to OECD Test Guidelines 414 and 416, respectively, synthetic amorphous silica (NM-200, nano-precipitated) did not cause prenatal developmental toxicity, perinatal toxicity or maternal toxicity or impair sexual maturation after being given in gavage doses up to the limit dose of 1000 mg/kg body weight and day. The following toxicokinetic data are taken into consideration for the extrapolation of the NOAEL of 1000 mg/kg body weight and day to a concentration in workplace air: the daily exposure of the animals in comparison with the 5 days per week exposure at the workplace (7:5) (only for the 2-generation study), the corresponding species-specific correction value for the rat (1:4), the assumed oral absorption (100%), the body weight (70 kg) and the respiratory volume (10 m^3^) of the person and the assumed 100% absorption by inhalation. The concentrations in the air calculated from the oral NOAEL for developmental toxicity and perinatal toxicity are 1750 and 2450 mg/m^3^, respectively. The concentrations in the air are 87 500 and 122 500 times as high as the MAK value of 0.02 mg/m^3^ R; the margins are thus sufficiently large. Even if oral absorption in animals were estimated to be only 20% (van der Zande et al. 2014), the 17 500-fold and 24 500-fold margins are sufficiently large for classification in Pregnancy Risk Group C. As a result, synthetic amorphous silica remains assigned to Pregnancy Risk Group C.

**Carcinogenicity. **No carcinogenic effects in humans can be derived from the data for synthetic amorphous silica. The tumours found in the lungs of rats in a 2-year study (Ernst et al. 2005; Kolling et al. 2008) are of only limited relevance for the evaluation because of the multiple applications, the high dose applied (30 intratracheal installations of 0.5 mg of amorphous silica at intervals of 2 weeks) and the limited number of histopathological investigations. The study used only one dose. The substance was applied repeatedly to compensate for the solubility of silica in the lungs, thereby ensuring that the lungs remained burdened by the particles for a longer period of time. In addition, a high lung burden was achieved with the dissolved SiO_2_. Therefore, the primary cause of tumour development was the severe inflammation and not the amorphous silica being tested. Reliable data are not available from epidemiological studies for this end point. Overall, the available studies are not suitable for drawing conclusions about carcinogenic effects in humans.

**Germ cell mutagenicity. **Synthetic amorphous silica did not induce mutations in vitro or in vivo. The DNA strand breaks and micronuclei reported by in vitro studies were not confirmed by in vivo studies. There are no studies that investigated germ cells. The data do not indicate that mutagenic effects occur and synthetic amorphous silica has not been classified in one of the categories for germ cell mutagens.

**Absorption through the skin. **There are no valid studies investigating absorption after dermal application. Earlier studies with the application of 10 000 mg/kg body weight and day to the skin of rabbits did not find evidence of the systemic uptake of silicon dioxide. With models and assuming standard conditions, a maximum uptake of 4 mg was calculated. As systemic toxicity is not predominant, synthetic amorphous silica has not been designated with an “H” (for substances which can be absorbed through the skin in toxicologically relevant amounts).

**Sensitization. **Positive clinical findings or positive results from experimental studies of the sensitizing effects of silica on the skin or airways are not available. For this reason, synthetic amorphous silica remains not designated with “Sh” or “Sa” (for substances which cause sensitization of the skin or airways).
